# Association of the Glycated Albumin‐to‐Glycated Haemoglobin Ratio With Mortality in Type 2 Diabetes: A Retrospective Cohort Analysis

**DOI:** 10.1002/edm2.70072

**Published:** 2025-07-01

**Authors:** Tomohito Gohda, Nozomu Kamei, Marenao Tanaka, Masato Furuhashi, Tatsuya Sato, Mitsunobu Kubota, Michiyoshi Sanuki, Risako Mikami, Koji Mizutani, Yusuke Suzuki, Maki Murakoshi

**Affiliations:** ^1^ Department of Nephrology Juntendo University Faculty of Medicine Tokyo Japan; ^2^ Department of Endocrinology and Metabolism Hiroshima Red Cross Hospital & Atomic‐Bomb Survivors Hospital Hiroshima Japan; ^3^ Institute for Clinical Research, NHO Kure Medical Center and Chugoku Cancer Center Hiroshima Japan; ^4^ Department of Cardiovascular, Renal and Metabolic Medicine Sapporo Medical University School of Medicine Sapporo Japan; ^5^ Department of Cellular Physiology and Signal Transduction Sapporo Medical University School of Medicine Sapporo Japan; ^6^ Department of Endocrinology and Diabetology NHO Kure Medical Center and Chugoku Cancer Center Hiroshima Japan; ^7^ Department of Advanced Biomaterials Graduate School of Medical and Dental Science, Institute of Science Tokyo Tokyo Japan; ^8^ Department of General Dentistry Graduate School of Medical and Dental Science, Institute of Science Tokyo Tokyo Japan

**Keywords:** chronic kidney disease, glycated haemoglobin‐to‐glycated albumin ratio, mortality, type 2 diabetes

## Abstract

**Introduction:**

The glycated albumin‐to‐glycated haemoglobin (GA/HbA1c) ratio is a potential marker of glycaemic variability; however, its association with adverse clinical outcomes in type 2 diabetes remains unclear. We aimed to determine whether the GA/HbA1c ratio is a better predictor of mortality and chronic kidney disease (CKD) progression than GA alone in type 2 diabetes.

**Methods:**

This retrospective cohort analysis included 571 Japanese participants with type 2 diabetes who were stratified into tertiles based on their GA/HbA1c ratio. Cox proportional hazards models assessed associations between the GA/HbA1c ratio and mortality or CKD progression (≥ 30% decline in the estimated glomerular filtration rate [eGFR]), adjusting for age, sex, urinary albumin‐to‐creatinine ratio, eGFR, body mass index, haemoglobin and serum albumin.

**Results:**

In this cohort, the median age was 67 years, and 53.9% were male. During the median follow‐up of 5.4 and 5.3 years for mortality and CKD progression, respectively, 40 (7.0%) participants died and 70 (12.3%) experienced CKD progression. For mortality, the GA/HbA1c ratio demonstrated a U‐shaped association: although both the lowest (T1) and highest (T3) tertiles showed higher mortality risks than the middle tertile (T2), this association was significant for only T3 (hazard ratio, 1.46; 95% CI, 1.05–2.04). Neither GA nor HbA1c alone was significantly associated with mortality. For CKD progression, GA alone showed a U‐shaped association, with both T1 and T3 exhibiting non‐significantly higher risks than T2. Neither the GA/HbA1c ratio nor HbA1c alone was associated with CKD progression.

**Conclusions:**

In individuals with type 2 diabetes, a higher GA/HbA1c ratio was associated with an increased risk of mortality but not with CKD progression. However, given the retrospective design and limited sample size, these findings should be interpreted with caution and confirmed in larger, prospective studies.

## Graphical abstract

The glycated albumin‐to‐haemoglobin A1c (GA/HbA1c) ratio independently predicted mortality in type 2 diabetes, showing a U‐shaped association. This ratio outperformed GA alone, highlighting its potential clinical utility as a prognostic marker.

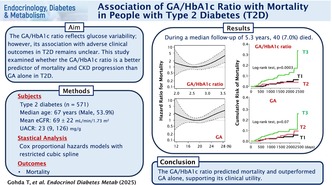

## 1 Introduction

Glycated haemoglobin (HbA1c) is the diagnostic clinical gold standard for monitoring glycaemic control in diabetes and reflects average blood glucose levels over the preceding 2–3 months [1]. In contrast, glycated albumin (GA) reflects the glycaemic status over a shorter 2–3‐week period [1]. Both markers are formed through a nonenzymatic reaction of their respective proteins. However, HbA1c can be influenced by red blood cell (RBC) lifespan, anaemia, uraemia and treatments such as erythropoiesis‐stimulating agents or iron supplementation [2]. These factors may lead to an underestimation of glycaemic control in individuals with chronic kidney disease (CKD), particularly those with diabetic kidney disease (DKD).

The GA‐to‐HbA1c (GA/HbA1c) ratio constitutes a useful marker of glycaemic variability and postprandial hyperglycaemia, independent of overall glycaemic control [3, 4, 5, 6, 7]. Glycaemic variability is associated with oxidative stress, endothelial dysfunction and increased risk of diabetic complications, including retinopathy and nephropathy [8, 9, 10, 11]. Independent of diabetes status, the GA/HbA1c ratio is associated with hippocampal atrophy and cognitive impairment [12, 13], which suggests a broader relationship between the GA/HbA1c ratio and vascular complications. Despite its association with these complications, the prognostic value of an elevated GA/HbA1c ratio for adverse outcomes, such as CKD progression and mortality, remains uncertain.

This study aimed to compare the associations of GA and the GA/HbA1c ratio with mortality and CKD progression in individuals with diabetes. Determining the prognostic utility of these markers may improve risk stratification and guide clinical management in this high‐risk population.

## 2 Materials and Methods

### 2.1 Study Design and Participants

This retrospective cohort study included 571 Japanese participants with type 2 diabetes. To observe the natural course of DKD, participants with diabetes were recruited from Kure Medical Center and Chugoku Cancer Center (Hiroshima, Japan) between July 1, 2014 and March 31, 2016. Of the 738 potential participants who were initially screened, 167 were excluded, primarily because of diabetes subtypes other than type 2 diabetes (*n* = 93), a baseline estimated glomerular filtration rate (eGFR) < 30 mL/min/1.73 m^2^ (*n* = 31), or a follow‐up period < 6 months (*n* = 39). The final study population comprised 571 participants (Figure S1).

The study was approved by the ethics committee of Kure Medical Center and Chugoku Cancer Center (approval number: 26–06) and was conducted in accordance with the principles of the Declaration of Helsinki and the Ethical Guidelines on Clinical Studies, as established by the Ministry of Health, Labour and Welfare of Japan. Written informed consent was obtained from all participants for study participation.

### 2.2 Sample Collection and Laboratory Measurements

GA was measured using an enzymatic method (LucicaGA‐L, Asahi Kasei Pharma, Tokyo, Japan) on a biochemical autoanalyser, whereas the HbA1c level was measured using an ion‐exchange high‐performance liquid chromatography (HPLC) analyser (Tosoh HLC‐723 G9, Tosoh Co., Tokyo, Japan). The serum creatinine level was measured using a standard enzymatic method. eGFR was calculated using formulas specific to the Japanese population, in conformance with the Japanese Society of Nephrology guidelines, as follows [14]:

$$\begin{array}{c} \text{eGFR}\;\left(\mathrm{mL}/\min/1.73\;m^{2}\right)=194\times {\left[\mathrm{age}\;\left(\text{years}\right)\right]}^{–0.287}\\ \times {\left[\text{serum creatinine}\;\left(\mathrm{mg}/\mathrm{dL}\right)\right]}^{–1.094}\times 0.739\;\text{(for women)} \end{array}$$

Urinary albumin and creatinine levels were quantified using immunonephelometry (N‐assay TIA Micro Alb; Nittobo Medical Co. Ltd., Fukushima, Japan) and enzymatic methods, respectively. The urinary albumin‐to‐creatinine ratio (UACR) was expressed in milligrams of albumin per gram of creatinine.

### 2.3 Adverse Outcomes (CKD Progression, Mortality and Composite Outcome)

CKD progression in participants with type 2 diabetes (or DKD progression) was defined as a ≥ 30% decline in eGFR from baseline during the follow‐up period, regardless of the baseline eGFR value. Participants were not required to meet the diagnostic criteria for CKD (i.e., eGFR < 60 mL/min/1.73 m^2^) at baseline. The 30% eGFR decline was used as the primary endpoint because only 35 (6.1%) participants experienced a ≥ 40% decline in eGFR from baseline at the end of the follow‐up period. Eligible participants were monitored every few months using blood tests. The first occurrence of a 30% decline in eGFR from baseline, confirmed by two consecutive blood tests taken at least 1 month apart, was recorded as the onset of CKD progression. The composite outcome was defined as CKD progression (30% decline in eGFR from baseline) and mortality.

### 2.4 Statistical Analysis

Continuous variables are expressed as mean ± standard deviation (SD) for normal distribution or median (interquartile range [IQR]) for skewed variables; categorical variables are presented as numbers and percentages. Participants were divided into three subgroups based on their baseline GA/HbA1c ratio, GA and HbA1c (tertiles 1, 2 and 3). Group characteristics are expressed as the mean ± SD, median (IQR), or frequency, as appropriate.

Between‐group comparisons were conducted using the one‐way analysis of variance or Kruskal–Wallis tests, as appropriate. Categorical variables were analysed using the chi‐square test. Spearman correlation analysis was used to assess relationships between the GA/HbA1c ratio and clinical parameters. Cox proportional hazards models incorporating penalised splines (via the pspline function in the *survival* package) were used to assess the nonlinear associations of the GA/HbA1c ratio, GA and HbA1c with CKD progression, mortality and their composite outcome. The models were adjusted for age, sex, eGFR, UACR, body mass index (BMI), haemoglobin and serum albumin. We used four degrees of freedom for smoothing. Knot positions in the penalised spline models were determined internally and not explicitly specified.

All analyses were performed using SAS v.9.4 (SAS Institute Inc., Cary, NC, USA) and R version 4.4.2 (The R Foundation for Statistical Computing, Vienna, Austria), with a two‐tailed *p* < 0.05 indicating statistical significance.

## 3 Results

### 3.1 Baseline Characteristics

Individuals were stratified into tertiles based on the GA/HbA1c ratio, and the clinical characteristics of these tertiles are summarised in Table 1. Individuals with a higher GA/HbA1c ratio were older, had a longer duration of diabetes and exhibited lower levels of BMI, alanine aminotransferase (ALT), diastolic blood pressure (BP), haemoglobin, serum albumin, eGFR, non–high‐density lipoprotein cholesterol (non–HDL‐C) and GA, whereas they had higher levels of high‐density lipoprotein cholesterol (HDL‐C). However, the three groups had similar HbA1c levels.

**TABLE 1:** Baseline characteristics according to glycated albumin‐to‐glycated haemoglobin ratio categorised as tertiles in participants with diabetes.

Characteristics	GA/HbA1c ratio (*n* = 571)			
	Tertile 1 (*n* = 192) < 2.46	Tertile 2 (*n* = 190) 2.46–2.80	Tertile 3 (*n* = 189) > 2.80	*p*
GA/HbA1c ratio	2.30 (2.18, 2.40)	2.64 (2.54, 2.71)	3.03 (2.89, 3.25)	by design
Age, yr	61 (51, 68)	67 (61, 73)	71 (66, 78)	< 0.001
Male, *n* (%)	90 (46.9)	115 (60.5)	109 (57.7)	0.02
BMI, kg/m^2^	27.0 (24.4, 29.8)	24.2 (22.2, 26.8)	22.8 (20.3, 25.5)	< 0.001
AST, U/L	22 (17, 28)	21 (17, 28)	22 (17, 27)	0.96
ALT, U/L	22 (15, 35)	20 (15, 28)	17 (13, 23)	< 0.001
Systolic BP, mmHg	138 ± 17	139 ± 18	140 ± 17	0.55
Diastolic BP, mmHg	81 ± 11	78 ± 12	76 ± 11	< 0.001
Hypertension, *n* (%)	141 (73.4%)	141 (74.2%)	135 (71.4%)	0.82
Prior CVD, *n* (%)	18 (9.4%)	26 (13.7%)	29 (15.3%)	0.20
Haemoglobin, g/dL	14.1 ± 1.6	13.7 ± 1.6	13.0 ± 1.8	< 0.001
Albumin, g/dL	4.4 (4.2, 4.7)	4.4 (4.1, 4.6)	4.3 (4.0, 4.5)	< 0.001
Diabetes duration, yr	10 (6, 18)	15 (9, 21)	18 (9, 27)	< 0.001
HbA1c, %	7.3 (6.7, 8.0)	7.2 (6.6, 7.8)	7.1 (6.4, 7.8)	0.12
GA, %	16.5 (15.2, 18.7)	18.8 (17.3, 20.9)	21.6 (19.8, 24.5)	< 0.001
eGFR, mL/min/1.73 m^2^	76.4 ± 22.9	67.1 ± 20.6	64.6 ± 19.6	< 0.001
UACR, mg/g	21 (9, 80)	31 (10, 169)	22 (9, 88)	0.14
HDL‐C, mg/g	48 (43, 55)	50 (43, 59)	54 (45, 65)	0.001
Non‐HDL‐C, mg/dL	129 (111, 157)	129 (111, 151)	119 (101, 141)	< 0.001
C‐reactive protein, mg/dL	0.11 (0.07, 0.20)	0.10 (0.06, 0.17)	0.09 (0.05, 0.19)	0.06

*Note:* Data are presented as mean ± SD, median (25th and 75th percentile) or *n* (percentage).

Abbreviations: ALT, alanine aminotransferase; AST, aspartate aminotransferase; BMI, body mass index; BP, blood pressure; CVD, cardiovascular disease; eGFR, estimated glomerular filtration rate; GA, glycated albumin; HbA1c, glycated haemoglobin; HDL‐C, high‐density cholesterol; SD, standard deviation; UACR, urine albumin‐to‐creatinine rate.

### 3.2 Correlation Between GA/HbA1c Ratio, GA and Clinical Markers

To investigate the factors that correlated with the GA/HbA1c ratio and GA, we analysed the correlation coefficients with various clinical parameters (Table 2). BMI demonstrated the strongest negative correlation with the GA/HbA1c ratio (*r* = −0.44, *p* < 0.0001), besides GA itself (*r* = 0.63, *p* < 0.0001). Other notable correlations included positive correlations with age (*r* = 0.40, *p* < 0.0001) and the duration of diabetes (*r* = 0.23, *p* < 0.0001), as well as negative correlations with the haemoglobin level (*r* = −0.29, *p* < 0.0001), eGFR (*r* = −0.24, *p* < 0.0001), ALT (*r* = −0.22, *p* < 0.0001), diastolic BP (*r* = −0.22, *p* < 0.0001) and serum albumin level (*r* = −0.20, *p* < 0.0001). HbA1c showed only a weak correlation with the GA/HbA1c ratio (*r* = −0.10, *p* = 0.01).

**TABLE 2:** Correlation coefficients of each variable with glycated albumin‐to‐glycated haemoglobin ratio and glycated albumin.

	GA/HbA1c ratio		GA	
	Correlation coefficient	*p*	Correlation coefficient	*p*
GA	0.63	< 0.0001	NA	NA
BMI	−0.44	< 0.0001	−0.16	0.0001
Age	0.40	< 0.0001	0.20	< 0.0001
Haemoglobin	−0.29	< 0.0001	−0.12	< 0.005
eGFR	−0.24	< 0.0001	−0.05	0.24
Duration	0.23	< 0.0001	0.21	< 0.0001
ALT	−0.22	< 0.0001	0.07	0.11
Diastolic BP	−0.22	< 0.0001	−0.15	< 0.0005
Albumin	−0.20	< 0.0001	−0.16	< 0.0005
HDL‐C	0.19	< 0.0001	0.05	0.22
Non‐HDL‐C	−0.18	< 0.0001	−0.02	0.70
C‐reactive protein	−0.10	0.01	0.03	0.50
Sex	0.10	0.01	0.01	0.77
HbA1c	−0.10	0.02	0.66	< 0.0001
Prior CVD	0.08	0.045	0.07	0.12
SBP	0.05	0.22	0.03	0.54
Hypertension	−0.02	0.62	−0.03	0.42
UACR	0.01	0.87	0.13	< 0.005
AST	0.004	0.92	0.11	0.01

Abbreviations: ALT, alanine aminotransferase; AST, aspartate aminotransferase; BMI, body mass index; BP, blood pressure; CVD, cardiovascular disease; eGFR, estimated glomerular filtration rate; GA, glycated albumin; HbA1c, glycated haemoglobin; HDL‐C, high‐density cholesterol; NA, not applicable; SD, standard deviation; UACR, urine albumin‐to‐creatinine ratio.

Similar to the GA/HbA1c ratio, the GA correlated with BMI (*r* = −0.16, *p* < 0.0001), age (*r* = −0.20, *p* < 0.0001), haemoglobin level (*r* = −0.12, *p* < 0.005), the duration of diabetes (*r* = 0.21, *p* < 0.0001), diastolic BP (*r* = −0.15, *p* < 0.0005) and the serum albumin level (*r* = −0.16, *p* < 0.0005). However, the strength of these correlations was generally weaker than that observed for the GA/HbA1c ratio. Furthermore, GA did not correlate with eGFR but showed only a weak positive correlation with UACR (*r* = 0.13, *p* < 0.002).

### 3.3 Associations Among the GA/HbA1c Ratio, GA and Clinical Outcomes in Univariate Analysis

During the median follow‐up period of 5.4 (IQRs: 4.6–6.0) and 5.3 (IQRs: 3.5–6.0) years for mortality and CKD progression, respectively, 40 individuals (7.0%) died from all causes, and 70 (12.3%) experienced CKD progression.

**FIGURE 1:**
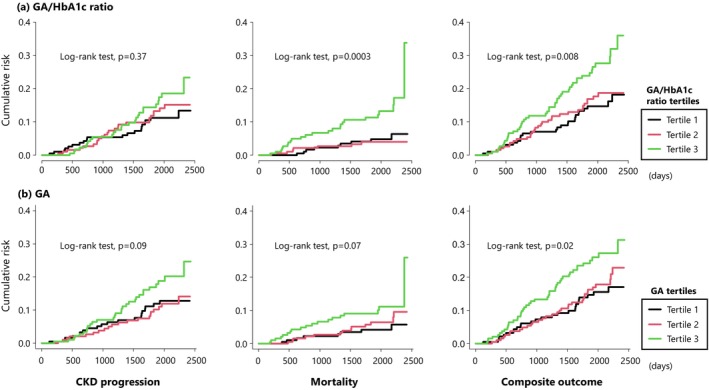
Cumulative risk of CKD progression, mortality and composite outcomes in individuals with diabetes mellitus, according to the tertile of (a) GA‐to‐HbA1c ratio and (b) GA at baseline. CKD, chronic kidney disease; GA, glycated albumin; HbA1c, glycated haemoglobin.

The cumulative incidence of adverse events was stratified by the tertiles of the GA/HbA1c ratio and GA (Figure 1). For the GA/HbA1c ratio, the cumulative incidence of mortality (*p* = 0.0003) and composite outcome (*p* = 0.008) were significantly higher in the highest tertile compared to those in the lowest or middle tertile. In contrast, the incidence of CKD progression did not differ across tertiles.

Regarding GA, the cumulative incidence of the composite outcome was significantly higher in the highest tertile than in those in the lowest and middle tertiles (*p* = 0.02). However, no significant differences were observed in the incidence of mortality or CKD progression across tertiles.

### 3.4 Associations Among the GA/HbA1c Ratio, GA and Clinical Outcomes in Multivariate Analysis

As shown in Table 3, compared to the reference group (T2) in the fully adjusted model (Model 4), individuals in the highest tertile of the GA/HbA1c ratio (T3) had a higher risk of mortality (HR: 2.95, 95% confidence interval: 1.24–6.98). Although the lowest tertile (T1) showed a higher HR relative to T2, this association was not statistically significant. HbA1c demonstrated a similar U‐shaped trend with mortality, although the differences in T1 and T3 did not reach statistical significance (Table S1). For GA alone, T3 showed a non‐significant trend towards increased mortality, whereas T1 was not associated with an increased mortality risk.

**TABLE 3:** Association of glycated albumin‐to‐glycated haemoglobin ratio and glycated albumin levels with CKD progression, mortality and composite outcome.

GA/HbA1c ratio	CKD progression, HR (95% CI)			Mortality, HR (95% CI)			Composite outcome, HR (95% CI)		
	T1	T2 (ref.)	T3	T1	T2 (ref.)	T3	T1	T2 (ref.)	T3
Model 1	0.83 (0.46, 1.51)	1	1.26 (0.72, 2.19)	1.28 (0.48, 3.44)	1	3.82 (1.65, 8.85)*	0.87 (0.52, 1.45)	1	1.70 (1.08, 2.68)^†^
Model 2	0.86 (0.46, 1.59)	1	1.25 (0.71, 2.19)	2.15 (0.78, 5.92)	1	3.63 (1.56, 8.45)*	1.05 (0.62, 1.79)	1	1.65 (1.04, 2.61)^†^
Model 3	0.96 (0.51, 1.79)	1	1.48 (0.83, 2.62)	2.22 (0.80, 6.10)	1	3.57 (1.53, 8.34)*	1.17 (0.68, 2.01)	1	1.80 (1.13, 2.88)^†^
Model 4	1.07 (0.57, 2.04)	1	1.11 (0.62, 2.01)	2.52 (0.91, 7.00)	1	2.95 (1.24, 6.98)^†^	1.34 (0.78, 2.33)	1	1.35 (0.83, 2.18)

GA	T1	T2 (ref.)	T3	T1	T2 (ref.)	T3	T1	T2 (ref.)	T3
Model 1	1.06 (0.57, 1.97)	1	1.73 (0.99, 3.05)	0.64 (0.27, 1.55)	1	1.61 (0.79, 3.26)	0.84 (0.50, 1.40)	1	1.57 (1.00, 2.46)^†^
Model 2	1.11 (0.59, 2.10)	1	1.77 (1.00, 3.12)^†^	0.86 (0.35, 2.10)	1	1.83 (0.90, 3.73)	0.98 (0.58, 1.66)	1	1.68 (1.07, 2.64)^†^
Model 3	1.23 (0.64, 2.37)	1	1.37 (0.78, 2.43)	0.88 (0.36, 2.15)	1	1.77 (0.87, 3.62)	1.07 (0.63, 1.82)	1	1.38 (0.88, 2.17)
Model 4	1.86 (0.95, 3.66)	1	1.71 (0.96, 3.05)	1.00 (0.41, 2.46)	1	1.63 (0.79, 3.34)	1.44 (0.83, 2.48)	1	1.50 (0.95, 2.36)

*Note:* Model 1, unadjusted; Model 2, age and sex; Model 3, Model 2 + UACR and eGFR; Model 4, Model 3 + BMI, haemoglobin and albumin. GA/HbA1c ratio: T1 (< 2.455), T2 (2.455–2.800), T3 (> 2.800); GA: T1 (≤ 17.6%), T2 (17.7%–20.8%), T3 (≥ 20.9%).

Abbreviations: ALT, alanine aminotransferase; AST, aspartate aminotransferase; BMI, body mass index; BP, blood pressure; CI, confidence interval; CKD, chronic kidney disease; CVD, cardiovascular disease; eGFR, estimated glomerular filtration rate; GA, glycated albumin; HbA1c, glycated haemoglobin; HDL‐C, high‐density cholesterol; HR, hazard ratio; ref., reference; SD, standard deviation; T, tertile; UACR, urine albumin‐to‐creatinine ratio.

^*^ *p* < 0.05.

^†^ *p* < 0.01.

Regarding CKD progression, GA exhibited a U‐shaped pattern, with both T1 and T3 showing a higher risk than T2; however, neither association was statistically significant in the fully adjusted model (Model 4). No significant associations were observed for CKD progression with either the GA/HbA1c ratio or HbA1c (Table 3 and Table S1). Similarly, none of the markers were significantly associated with the composite outcome (Table 3 and Table S1).

**FIGURE 2:**
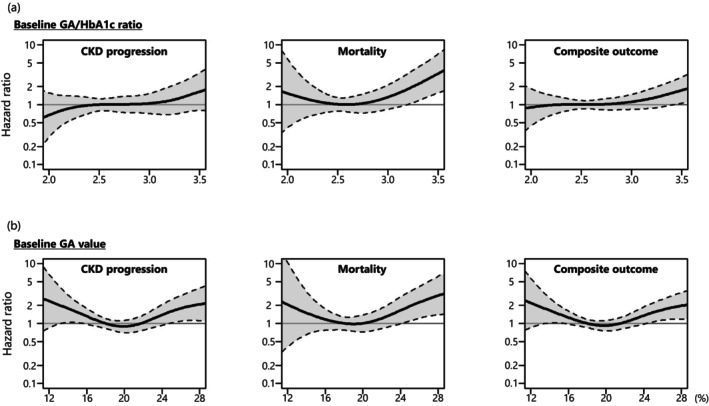
HRs for CKD progression, mortality and their composite outcome (CKD progression + mortality) according to (a) GA/HbA1c ratio and (b) GA at baseline, which was estimated using multivariable Cox proportional hazards models with restricted cubic splines, adjusted for age, sex, eGFR, UACR, BMI, haemoglobin and serum albumin. Solid line: HR; dashed line: 95% confidence interval. The reference values of the GA/HbA1c ratio and GA were 2.55% and 18%, respectively. BMI, body mass index; CKD, chronic kidney disease; eGFR, estimated glomerular filtration rate; GA, glycated albumin; HbA1c, glycated haemoglobin; HR, hazard ratio; UACR, urine albumin‐to‐creatinine rate.

The results of restricted cubic spline analyses are presented in Figure 2. The GA/HbA1c ratio showed a right‐skewed U‐shaped curve with respect to mortality (Figure 2a), which suggests a potential increase in risk at both the lower and higher ends of the distribution. A similar pattern was observed for GA (Figure 2b). For CKD progression and the composite outcome, GA showed a U‐shaped relationship, whereas the GA/HbA1c ratio did not exhibit clear trends.

## 4 Discussion

Our study provides novel insights by demonstrating that, in individuals with type 2 diabetes, an elevated GA/HbA1c ratio is significantly associated with mortality even after adjustment for relevant covariates, whereas neither GA nor HbA1c alone was independently associated with mortality.

The biological mechanisms that link the GA/HbA1c ratio to mortality remain unclear; however, several hypotheses have been proposed. A high GA/HbA1c ratio possibly reflects increased glycaemic variability and postprandial hyperglycaemia, both of which are associated with oxidative stress, endothelial dysfunction and atherosclerotic progression—which are key drivers of diabetes‐related vascular complications and, ultimately, higher mortality risk [7, 8, 9]. Moreover, the GA/HbA1c ratio may be affected by non‐glycaemic factors, such as anaemia, inflammation and shortened RBC lifespan [15, 16, 17], all of which can lower HbA1c independently of glycaemic control and have individually been linked to poor outcomes [18, 19]. Therefore, an elevated GA/HbA1c ratio may reflect a combination of adverse glycaemic and non‐glycaemic conditions that contribute to mortality. Conversely, a low GA/HbA1c ratio may reflect chronically elevated glucose levels with relatively low variability or potentially even unrecognised hypoglycaemia. Although the mechanisms underlying increased mortality at the lower end of the GA/HbA1c spectrum are less understood, this finding indicates the possibility that both excessive glycaemic fluctuation and sustained hyperglycaemia without adequate variability may be detrimental to the clinical outcomes of individuals with type 2 diabetes.

Despite its stronger correlations with established CKD risk factors—such as lower BMI, haemoglobin level, serum albumin level and eGFR—the GA/HbA1c ratio was not significantly associated with CKD progression, whereas GA alone exhibited a U‐shaped relationship with CKD progression, which is consistent with the findings of previous studies [20]. This apparent discrepancy may be explained by the heightened sensitivity of the GA/HbA1c ratio to non‐glycaemic factors, such as malnutrition, anaemia and chronic inflammation [21, 22, 23, 24, 25], all of which are highly prevalent in advanced CKD and contribute to CKD progression. Although these associations enhance the ability of the GA/HbA1c ratio to capture overall health status, they may dilute its specificity as a marker for glycaemic burden and reduced kidney function. In contrast, despite being less influenced by these non‐glycaemic factors, GA itself may better reflect glycaemic exposure that is relevant to renal outcomes. Therefore, although the GA/HbA1c ratio may serve as a useful prognostic marker for mortality in individuals with type 2 diabetes, caution is warranted when interpreting its utility for predicting CKD progression. The divergence in its associations with mortality versus CKD progression underscores the importance of clinical context in evaluating this biomarker.

This study has some limitations. First, the sample size was not determined based on a priori power calculations, as the analysis was retrospective and based on a pre‐existing cohort that was originally designed to evaluate the long‐term clinical course of individuals with type 2 diabetes. Nevertheless, the relatively long follow‐up period and the number of observed events (CKD progression and mortality) enabled statistically robust analyses. Second, cause‐of‐death data were unavailable, which prevented sensitivity analyses based on cause‐specific mortality. Third, serial measurements of albuminuria were not obtained, and CKD progression was defined solely by a decline in eGFR. Although this definition captures clinically relevant loss in kidney function, the absence of albuminuria‐based endpoints is a notable limitation. Fourth, the study was conducted at a single centre and included only Japanese individuals with diabetes, which may limit the generalisability of the findings. Finally, both GA and HbA1c were measured only at baseline, which precluded an evaluation of temporal trends or variability in glycaemic control. As with all observational studies, residual confounding owing to unmeasured variables cannot be excluded.

In conclusion, in individuals with type 2 diabetes, a higher GA/HbA1c ratio was independently associated with an increased risk of mortality, whereas no significant association was observed with CKD progression. This association may be partly influenced by non‐glycaemic factors such as anaemia, malnutrition and inflammation, which can affect the GA/HbA1c ratio. Rather than serving as a specific predictor of CKD progression, the GA/HbA1c ratio may reflect broader aspects of overall health status. These findings are in line with previous reports in the general population, individuals with non‐alcoholic fatty liver disease and those on dialysis and may suggest a potential prognostic role for the GA/HbA1c ratio across diverse clinical contexts [26, 27, 28]. However, given the retrospective design and limited sample size of the current study, these findings should be interpreted with caution. Further large‐scale prospective studies are warranted to validate these associations and elucidate the underlying mechanisms.

## Author Contributions

**Tomohito Gohda:** conceptualization, formal analysis, methodology, project administration, writing – original draft, writing – review and editing. **Nozomu Kamei:** data curation, resources. **Marenao Tanaka:** data curation, formal analysis. **Masato Furuhashi:** data curation, methodology. **Tatsuya Sato:** data curation. **Mitsunobu Kubota:** data curation, resources. **Michiyoshi Sanuki:** data curation, resources. **Risako Mikami:** data curation. **Koji Mizutani:** data curation, methodology. **Yusuke Suzuki:** conceptualization, supervision, writing – review and editing. **Maki Murakoshi:** conceptualization, data curation, formal analysis, writing – review and editing.

## Conflicts of Interest

The authors declare no conflicts of interest.

## Supporting information


**Figure S1.** Screening and selection of study participants.

**Table S1.** Association of glycated haemoglobin with CKD progression, mortality and composite outcome.

## Data Availability

The data that supports the findings of this study are available in the Supporting Information of this article.
