# Supplementary material for: Association of the Glycated Albumin‐to‐Glycated Haemoglobin Ratio With Mortality in Type 2 Diabetes: A Retrospective Cohort Analysis
Source: Endocrinol Diabetes Metab. 2025 Jul 1;8(4):e70072. doi: 10.1002/edm2.70072 (PMC12209710; doi:10.1002/edm2.70072)
Supplement: Supplementary file 1 — Figure S1. Screening and selection of study participants. [file EDM2-8-e70072-s001.pptx]

## Slide 1
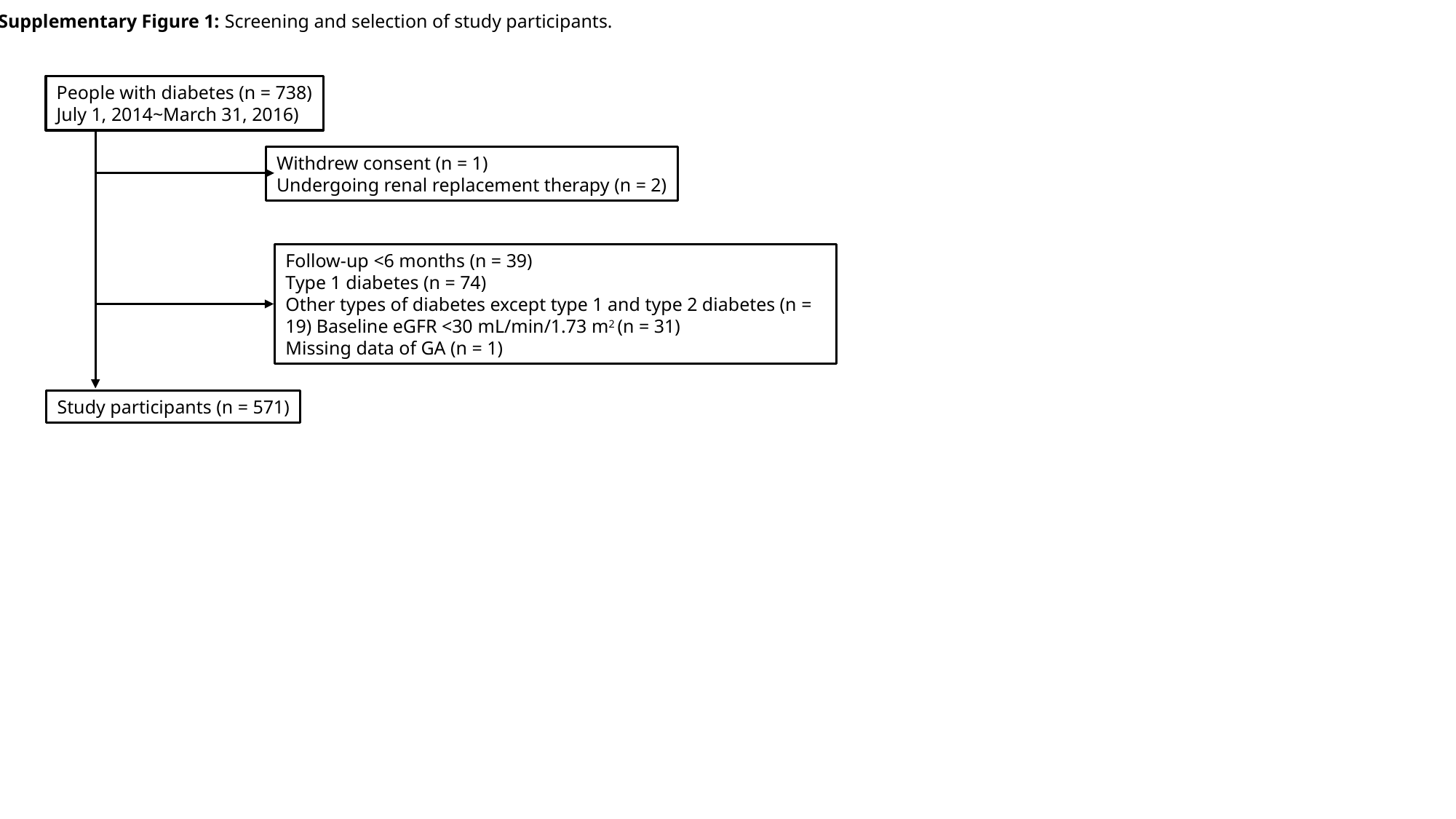

Supplementary Figure 1: Screening and selection of study participants.
People with diabetes (n = 738)
July 1, 2014~March 31, 2016)
Withdrew consent (n = 1)
Undergoing renal replacement therapy (n = 2)
Follow-up <6 months (n = 39)
Type 1 diabetes (n = 74)
Other types of diabetes except type 1 and type 2 diabetes (n = 19) Baseline eGFR <30 mL/min/1.73 m2 (n = 31)
Missing data of GA (n = 1)
Study participants (n = 571)
